# A Solar Cell Compatible Super-Wideband Flexible Transparent Antenna with Enhanced Axial Ratio

**DOI:** 10.3390/mi16111291

**Published:** 2025-11-18

**Authors:** Nouman Rasool, Shuqi Yang, Chen Chen, Zhengming Tang, Kama Huang, Jinwei Gao

**Affiliations:** 1 School of Electronic Information Engineering, China West Normal University, Nanchong 637002, China; engr_nouman77@yahoo.com (N.R.); shuqiyang@stu.cwnu.edu.cn (S.Y.); zhengmtang@163.com (Z.T.); 2 School of Physics and Astronomy, China West Normal University, Nanchong 637002, China; 212023070200021@stu.cwnu.edu.cn; 3 College of Electronic and Information Engineering, Sichuan University, Chengdu 610207, China; kmhuang@scu.edu.cn

**Keywords:** transparent antennas, super-wideband antenna, circular polarization

## Abstract

A super-wideband transparent antenna (SWTA) with wide axial ratio bandwidth (ARBW) featuring an enhanced ground plane and microstrip feeding is proposed. The antenna has planar dimensions of 0.20$\lambda_{0}$ × 0.20$\lambda_{0}$ × 0.003$\lambda_{0}$ at its lowest frequency of 1.33 GHz. The antenna is fabricated from a combination of PET and metal oxide thin films, which together enable its flexibility and transparency. The L-shaped strips attached to the ground perturb the electric field in the slot, exciting a pair of orthogonal modes and resulting in circular polarization. The proposed antenna demonstrate high performance with an impedance bandwidth of 182% (1.33–28.52 GHz), an axial ratio bandwidth of 66% (3.88–7.73 GHz), and attain a peak gain of 11.5 dBi. Moreover, with an optical transparency exceeding 90%, this design is a flexible, transparent, super-wideband (SWB) antenna capable of high data rates, easy integration, and beyond-visual-line-of-sight (BVLOS) operations.

## 1. Introduction

With the rapid growth of the Internet of Things (IoT) and the widespread pursuit of green and clean energy, the requirement for transparent antennas (TAs) becomes evident. Traditional wideband antennas are typically fabricated using various opaque substrates and conducting materials, e.g., FR-4, Rogers 5880, and copper or silver, respectively. Likewise, the implementation of TAs integrable into automotive systems have become a critical priority for applications encompassing vehicle-integrated solar cells, smart mobility interfaces, and multifunctional windshield technologies [1,2,3,4]. In response to the growing demand for advanced wireless communication, a major focus of research and development is the design and fabrication of high-performance, portable, and conformal flexible antennas. Consequently, the use of transparent conductive films on transparent substrates for microwave components has garnered significant research attention [5,6], leading to a rising demand for SWBTA antennas fabricated on flexible substrates.

While multiple materials have been explored for the production of transparent flexible antennas, indium tin oxide (ITO) is the most widely utilized due to its favorable combination of high transparency and good conductivity [7,8]. However, antennas fabricated from transparent conductive films exhibit lower radiation gain and efficiency compared to their metallic counterparts. Although metal mesh or grid structures [9,10] provide high conductivity and radiation efficiency for transparent antennas, their pronounced shadow effect often degrades the efficiency of integrated solar cells. With the development of circularly polarized (CP) antennas [11,12,13,14], wireless communication systems have achieved remarkable advancements [15,16]. These antennas offer significant benefits, including the ability to reduce multipath interference and fading effects, as well as to prevent polarization mismatch caused by Faraday rotation [17]. A key metric for evaluating the practicality of CP antennas is the axial ratio bandwidth (ARBW), defined as the frequency range over which the axial ratio remains below a specific threshold, typically 3 dB. Antennas with a wide ARBW [18] provide substantial advantages. They enhance system robustness against frequency variations; enable a single antenna to cover multiple frequency bands, thereby simplifying the system structure; and maintain high polarization matching efficiency across a wide band, which improves overall link performance. Despite these benefits, circularly polarized transparent antennas (CPTAs) have been rarely reported in the recent literature [1], primarily due to material limitations, structural complexity, and the challenge of ensuring high optical transparency. However, the ongoing evolution of wireless technology and its demand for high-data-rate communication are now driving the need for CP antennas with wide ARBW [19,20].

Several wideband [21,22,23,24] and ultra-wideband (UWB) [25,26] transparent antennas have been reported in the recent literature; however, the antennas in [22,23] utilize water and plexiglass materials which require an enclosure to contain the water, resulting in a complex fabrication and bulky structure. Additionally, a few transparent antenna utilize metal mesh, PET, and glass substrates to accommodate the antenna weight, but offer narrower bands that are larger in size [21], have a dual feed, and are lacking in flexibility [1,24,25,26]. However, TAs in [24,25] achieved broadband characteristics at the cost of compromised peak gain. Similarly, the TAs in [27] have a flexible substrate, though they only achieved narrowband CP. The proposed design offers a wider IBW that is more compact in size and with a wideband CP compared to the antenna in [1].

Super-wideband (SWB) [28,29] technology possesses a large bandwidth and extensive data capacity, making it suitable for rapid audio and video transmission. Compared to UWB, SWB technology delivers enhanced channel capacity, superior timing precision, and higher image resolution. Moreover, wide bandwidth has become indispensable, proving advantageous for short-range communication systems and a cornerstone for next-generation telecommunications.

This paper proposes a compact, flexible, and transparent super-wideband antenna with wide axial ratio bandwidth. Circular polarization is achieved by introducing an asymmetric perturbation in the resonant slot via an attached L-shaped strip. The antenna features a simple, compact geometry that facilitates fabrication. The final design exhibits high mechanical flexibility and demonstrates significant potential for diverse application platforms, including glass windows, photovoltaic cells for hybrid RF/solar energy harvesting, and future-generation Internet of Things (FG-IoT) devices.

## 2. Antenna Design and Operating Principle

### 2.1. The Geometry of Antenna

**Figure 1:**
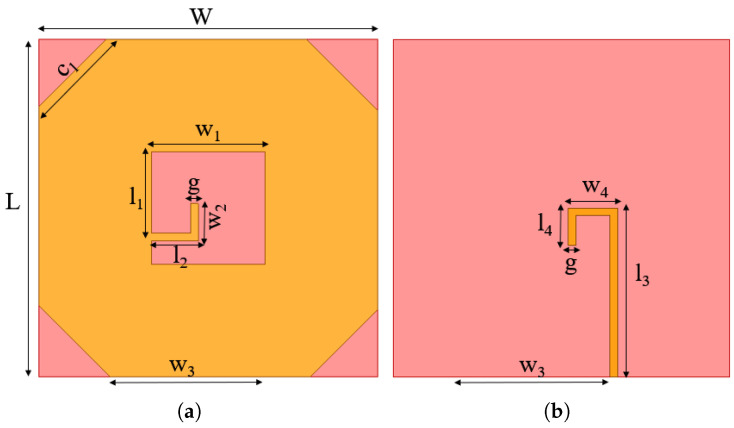
Basic antenna structure: (**a**) frontview; (**b**) back side.

Figure 1 describes the basic antenna structure, and the specific design parameters are shown in Table 1. The proposed antenna comprises a square radiating slot with an L-shaped strip and microstrip feed line. To realize the CP, the electric field slot is perturbed by an L-shaped strip connected to the ground plane. The proposed design utilized a 50 ohm microstrip line with an inverted L-shape extension towards the center of the radiating slot to achieve circular polarization.

The structural parameters listed in Table 1 are the result of an iterative design evolution driven by specific electromagnetic objectives. The initial dimensions were derived from fundamental principles and subsequently optimized through full-wave electromagnetic simulation (e.g., HFSS/ADS). The optimization process is guided by minimizing the return loss S_11_ at the center frequency. To address the inherent physical limitations of transparent materials in balancing optical transparency and electrical conductivity, an ITO substrate with low sheet resistance is selected as the material platform for fabricating transparent antenna. The radiation efficiency of a transparent antenna decreases with higher ITO sheet resistance, while its optical transparency generally improves. To balance this trade-off, an ITO material with a sheet resistance of 5 Ω/sq was selected. This value was chosen to maximize radiation efficiency while maintaining a high optical transparency of over 90%. The antenna is fabricated on a flexible PET substrate (relative permittivity = 3.7; dielectric loss tangent = 0.018). Meanwhile, PET with a thickness of 0.8 mm is utilized due to its excellent mechanical robustness, high optical transparency, low reflection loss, and high frequency applicability. The design basis for key parameters is initiated with the substrate size and the ground plane dimensions (W, L = 45 mm). The initial size was chosen to be electrically large at the lower band edge to ensure a stable ground effect and was later finalized for optimal performance. The microstrip feed line (length l_3_ = 22.5 mm; width w_3_ = 19.75 mm) was designed for a 50 Ω characteristic impedance. This was calculated using standard microstrip line formulas for the given substrate parameters (PET, $\varepsilon_{r}$ ≈3.7, height h = 0.8 mm). The optimized value was set to achieve the desired super-wideband impedance matching by optimizing the model in HFSS (version 2022 R2). The electrical length of the radiator at the lower ARBW frequency (3.88 GHz, $\lambda_{0}$ ≈77.3 mm) is 0.58λ, which is approximately λ/2, a fundamental resonance condition.

### 2.2. Antenna Evolution

**Figure 2:**
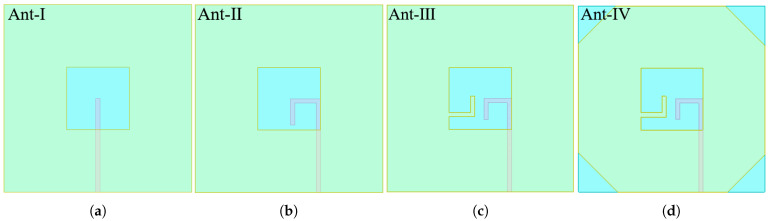
Antenna evolution steps, Ant-I to Ant-IV: (**a**) Ant-I, (**b**) Ant-II, (**c**) Ant-III, (**d**) Ant-IV.

**Figure 3:**
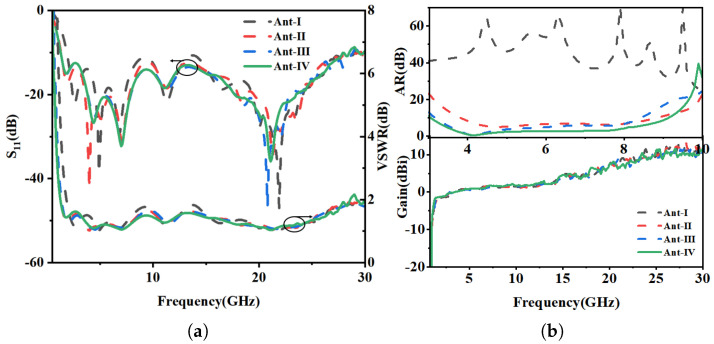
Simulation results of antenna evolution for Ant-I to Ant-IV. (**a**) Return loss and VSWR. (**b**) Axial ratio and peak gain.

Initially, the Ant-I was fed by a conventional 50 Ω microstrip line (MSL) of length $l_{3}$. To excite circular polarization (CP), the feeding structure was modified by extending an inverted L-strip from the MSL. The MSL, along with the inverted L-strip, was offset by 7 mm from the slot’s center, towards its right edge, in Ant-II to perturb the field distribution. While this perturbation successfully excited orthogonal degenerate modes, evidenced by a significant drop in the axial ratio (AR) from 60 dB to 10 dB, to achieve robust CP operation, a second L-shaped stub was introduced from the left side of the slot in Ant-III. This final modification creates the necessary asymmetric perturbation within the resonant cavity, unbalancing the electric field to produce two orthogonal modes with a 90° phase shift, thereby realizing a functional CP wave. A final design Ant-IV optimization involved chamfering the four corners of the ITO radiator as shown in Figure 2. This improvement is attributed to the dual effect of the chamfers resulting enhanced gain through surface wave suppression and, thereby improving the phase balance between orthogonal modes over a wider frequency range that results in an enhanced axial ratio bandwidth. The final chamfering in Ant-IV further optimized the current path length, which can be quantified by the phase constant (β). The improved phase balance (ΔΦ) across the bandwidth is given by (1)

(1) $$\Delta \Phi =\beta^{*}\Delta L$$

where Δ*L* is the effective change in the current path due to the chamfers, leading to a more stable phase relationship between modes over a wider frequency range, thus enhancing the axial ratio bandwidth (ARBW) depicted in Figure 3.

Furthermore, throughout the design evolution from Ant-I to Ant-IV, a Voltage Standing Wave Ratio (VSWR) < 2 was consistently maintained. This confirms that the impedance matching was preserved despite structural modifications, validating the design methodology.

### 2.3. Equivalent Circuit Modelling of the Proposed Antenna

**Figure 4:**
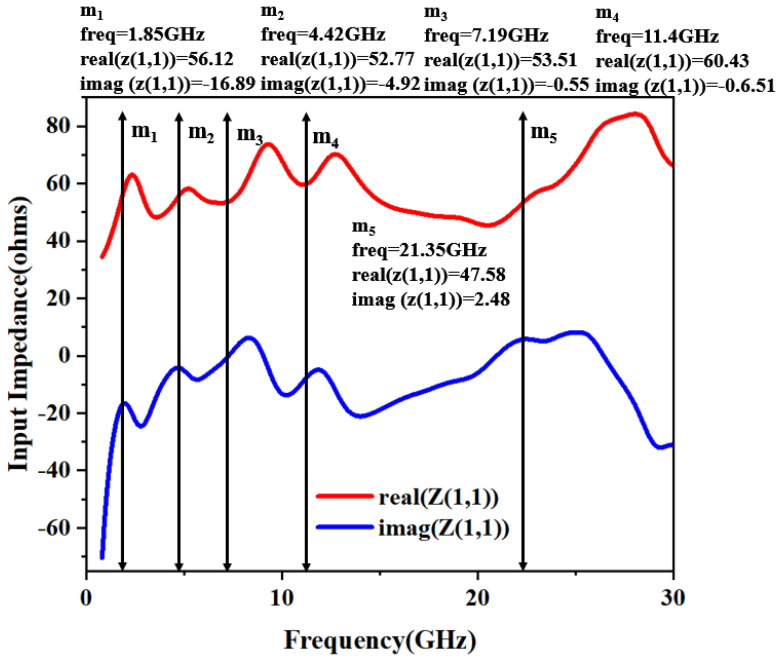
Real and imaginary parts of input impedance versus frequency.

The equivalent circuit model of the designed antenna is implemented using the Advanced Design System (ADS), as illustrated in Figure 4. The model was derived from the reflection coefficient (S_11_) response, where each resonance (identified by an attenuation dip below −10 dB) is represented by a parallel RLC circuit. Five distinct resonant modes are modeled within the operational band from 1.33 to 28.52 GHz. Markers m_1_ to m_5_ in Figure 4 denote these resonant frequencies. At these points, the input impedance has a real part close to 50 Ω and an imaginary part near 0 Ω, confirming excellent impedance matching.

**Figure 5:**
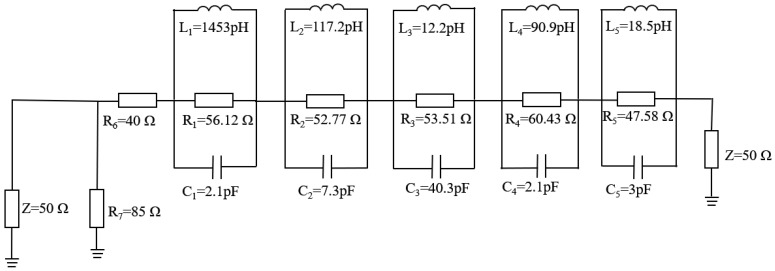
Equivalent circuit model in ADS.

The equivalent model derived from the data points of the input impedance is shown in Figure 5. The lumped element values (i.e., *R*, *L*, *C*) of the equivalent circuit can be calculated initially by using Equations (2)–(4) considering the bandwidth and resonant frequency matching to 50 Ω impedance matching condition. The lumped component values are finely adjusted in the ADS software (version 2021)to achieve the desired SWB characteristics. The impedance is represented by five parallel RLC cells that are connected in series and they resonate at respective resonant frequencies. Among them, *L* is the inductance, *C* is the capacitance, *f* is the resonant frequency, and imag(Z_11_) is the imaginary part of the impedance.

(2) $$L=\frac{\mathrm{imag}(Z_{11})}{2\pi f}$$

(3) $$C=\frac{1}{{\left(2\pi f\right)}^{2}L}$$

(4) $$f=\frac{1}{2\pi \sqrt{LC}}$$

**Figure 6:**
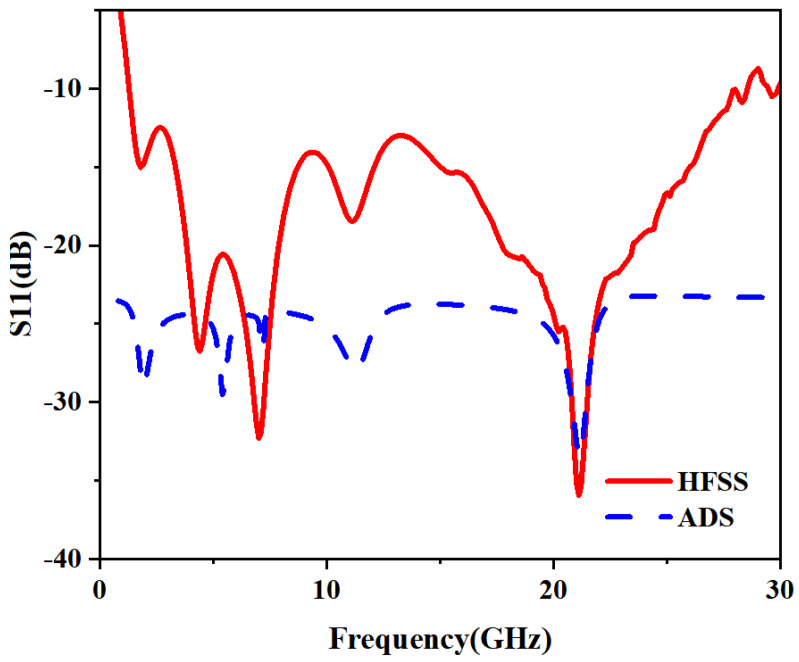
Comparison of reflection coefficient obtained from HFSS and ADS.

Figure 6 illustrates the comparison between the reflection coefficients obtained from HFSS and ADS. As illustrated in Figure 6, there is a small shift between the results obtained from the HFSS and ADS simulations. This discrepancy arises because the ADS circuit model, composed of idealized lumped components (*R*, *L*, *C*), is an approximation of the complex electromagnetic behavior captured by HFSS. The component values in ADS were optimized to fit the overall response, which can result in slightly shifted resonant frequencies compared to the full wave solution. Notably, the ADS model demonstrates superior impedance matching, achieving a return loss better than −30 dB.

### 2.4. Antenna Current Distribution and CP Realization

**Figure 7:**
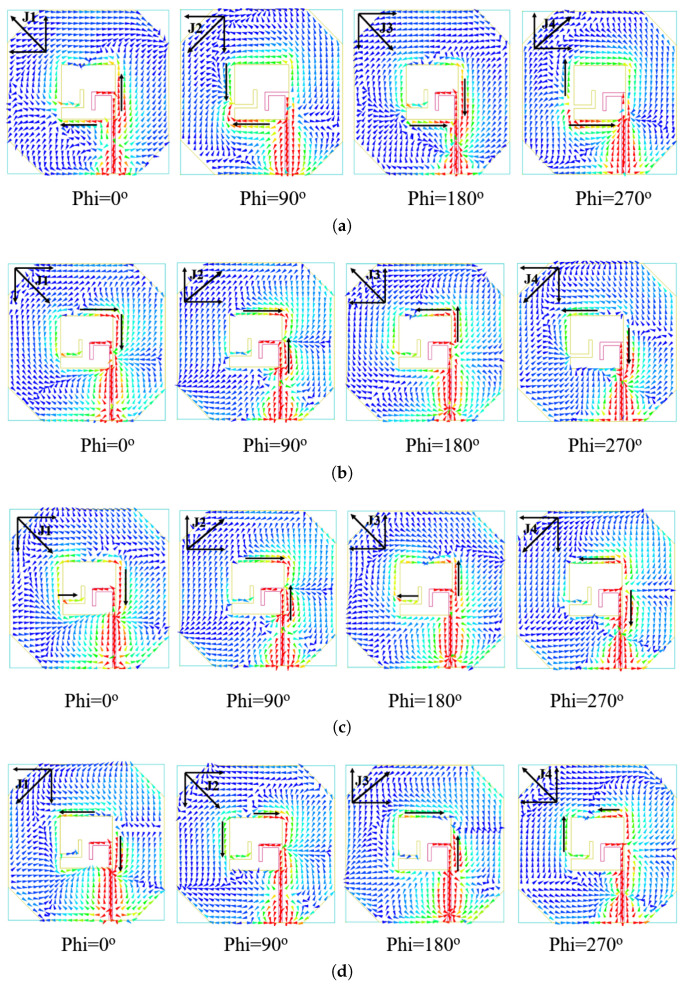
Current distribution of proposed antenna: (**a**) at 4.3 GHz; (**b**) at 5.8 GHz; (**c**) at 6.5 GHz; (**d**) at 7 GHz.

Figure 7 illustrates the principle of circular polarization through simulated surface current distributions at distinct time phases (0°, 90°, 180°, 270°) for frequencies of 4.3, 5.8, 6.5, and 7 GHz. The sequence at 4.3 GHz demonstrates a clear counterclockwise rotation of the dominant current vector. Initially, at ωt = 0°, the current flows primarily along the +Y and −X axes, forming vector J_1_. Later, at ωt = 90°, the current shifts to the −X and −Y axes, forming vector J_2_. Furthermore, at ωt = 180°, it aligns with the −Y and +X axes, forming vector J_3_. Finally, at ωt = 270°, the current flows along the +X and +Y axes, forming vector J_4_. This sequential, counterclockwise rotation of the current vector observed from the +z direction confirms the generation of a right-hand circular polarization (RHCP) wave. This characteristic rotational behavior is consistently observed across the antenna’s operational bandwidth.

### 2.5. Add a Solar Cell to the Antenna

**Figure 8:**
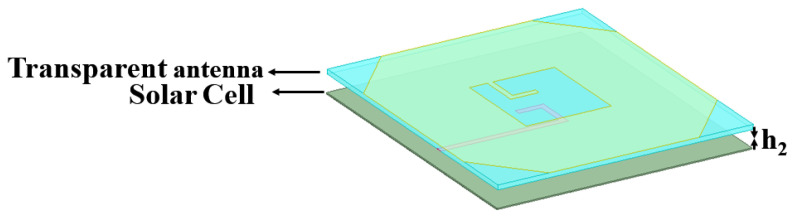
Schematic of solar cell underneath the transparent antenna.

**Figure 9:**
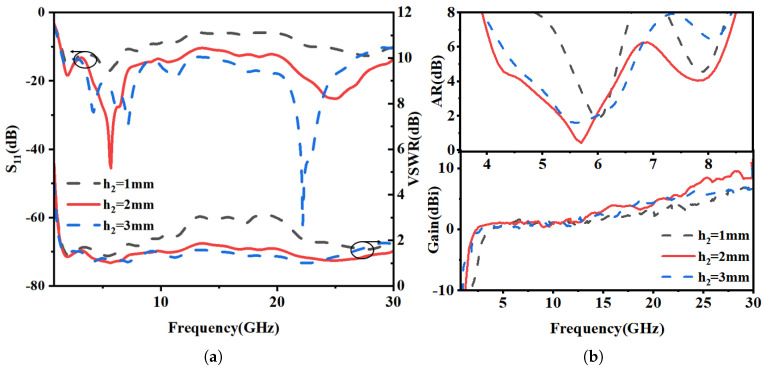
Antenna performance with solar cell at various separation distances (h_2_). (**a**) Return loss and VSWR. (**b**) Axial ratio and peak gain.

The effect of integrating a solar cell on antenna performance was investigated. A cell with a thickness of 0.2 mm ($\varepsilon_{r}$ = 1.5, µ = 1) was placed beneath the transparent antenna, and its vertical position was varied at heights of h_2_ = 1 mm, 2 mm, and 3 mm. A schematic of the solar cell integrated beneath the transparent antenna (TA) is provided in Figure 8. The superior optical transparency of transparent antennas (TAs) over conventional grid and mesh structures maximizes light incidence on the underlying solar cell, thereby minimizing the shadow effect and facilitating optimal solar conversion efficiency. Figure 9 compares and analyzes the return loss (S_11_), VSWR, axial ratio (AR), and gain characteristics of the antenna and the solar cell at different separation heights.

Figure 9a shows the reflection coefficient (S_11_) and VSWR for different solar cell separation heights (h_2_). At a close proximity of h_2_ = 1 mm, strong near-field coupling severely detunes the antenna, degrading the return loss to above −8 dB and indicating significant impedance mismatch. This coupling effect diminishes at larger separations (h_2_ = 2 mm and 3 mm), where the impedance bandwidth is largely maintained. The VSWR data further corroborates the restoration of proper impedance matching at these greater heights.

As shown in Figure 9b, the solar cell acts as a parasitic element, perturbing the antenna’s surface current distribution upon integration. This perturbation disrupts the critical amplitude and phase balance of the orthogonal modes, degrading the circular polarization purity and narrowing the axial ratio bandwidth. Concurrently, the solar cell absorbs radiative energy, leading to a slight reduction in gain, as evidenced by the peak gain of 6.31 dBi in its presence.

## 3. Fabrication and Measurement of Antenna

### 3.1. Fabrication and Transparency

**Figure 10:**
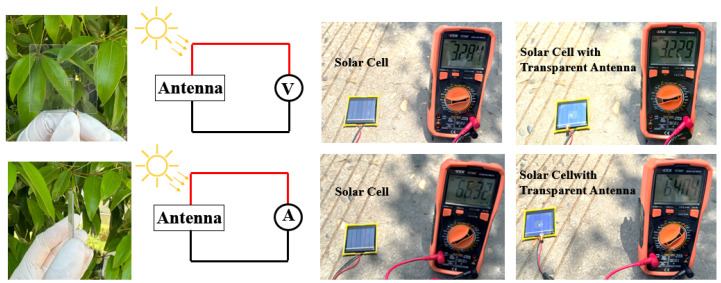
Transparent antenna prototype and its influence on solar cell output.

In transparent antenna design, metal oxide films like indium tin oxide (ITO) and aluminum-doped zinc oxide (AZO) are preferred radiating materials due to their favorable trade-off between electrical conductivity and optical transparency. The antenna was fabricated by laser-patterning a commercially available ITO-coated PET sheet, which was then laminated onto a dielectric substrate. The antenna’s impact on solar cell performance was quantified by measuring the cell’s current and voltage before and after antenna integration. The results show a negligible degradation in output, with the retained voltage at 98.3% and the retained current at 96.2% of their original values. This corresponds to minimal power loss, demonstrating that the antenna has an insignificant effect on the solar cell’s operational efficiency, as summarized in Figure 10.

Finally, the optical transparency of the fabricated antenna was measured with a TMD-II transparency tester, confirming a transparency level of 90%. These results collectively validate that the proposed antenna possesses SWB chrematistics, with high transparency and minimal impact on solar energy harvesting.

### 3.2. Antenna Measurement and Radiation Characteristics

**Figure 11:**
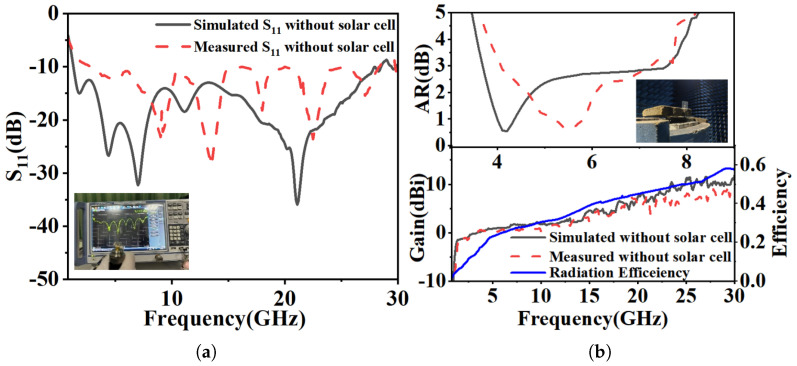
Simulated and measured results without solar cell. (**a**) Return loss. (**b**) Axial ratio and peak gain.

To validate the proposed design and simulation results, it was measured using a Rohde & Schwarz (ZNB 40) Vector Network Analyzer, whereas the far-field parameters were measured in an anechoic chamber, as shown in Figure 11.

The gain and axial ratio of the circularly polarized antenna under test (AUT) were characterized in a fully anechoic chamber using a far-field transmission setup. To measure the peak realized gain, a standard-gain horn antenna was used as the source, and the transmission coefficient (S_21_) was recorded. The AUT was rotated around its boresight axis to find the maximum received power, and the gain was calculated using the gain-transfer (comparison) method. The axial ratio was determined using a rotating linear source antenna. At each frequency point, the source was rotated through 360°, and the received power by the AUT was recorded. The axial ratio (*AR*) was then calculated from (5)

(5) $$AR\left[\mathrm{dB}\right]=10\log_{10}\left(\frac{P_{\max}}{P_{\min}}\right)$$

Figure 11a shows close agreement between the simulated and measured impedance bandwidth (IBW), with the simulated result spanning from 1.33 to 28.52 GHz (182% fractional bandwidth). Figure 11b compares the simulated and measured radiation performance. The measured axial ratio bandwidth (ARBW) is in good agreement with the simulation, and the measured peak gain is close to the simulated value of 11.5 dBi.

**Figure 12:**
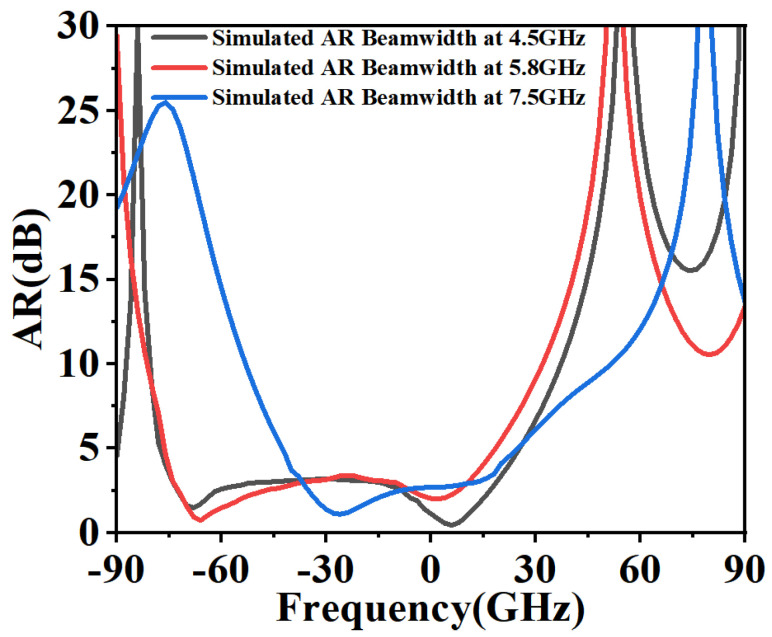
Axial ratio beamwidth for RHCP at 4.5 GHz, 5.8 GHz, and 7.5 GHz.

**Figure 13:**
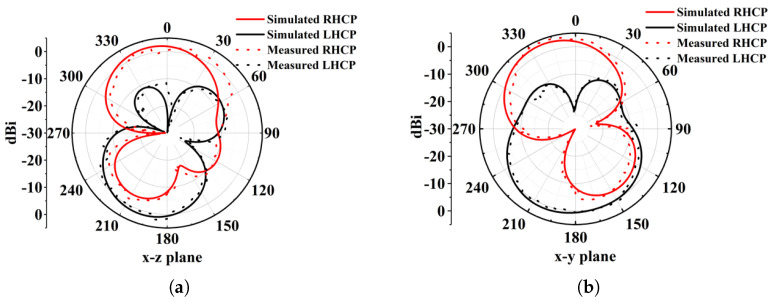

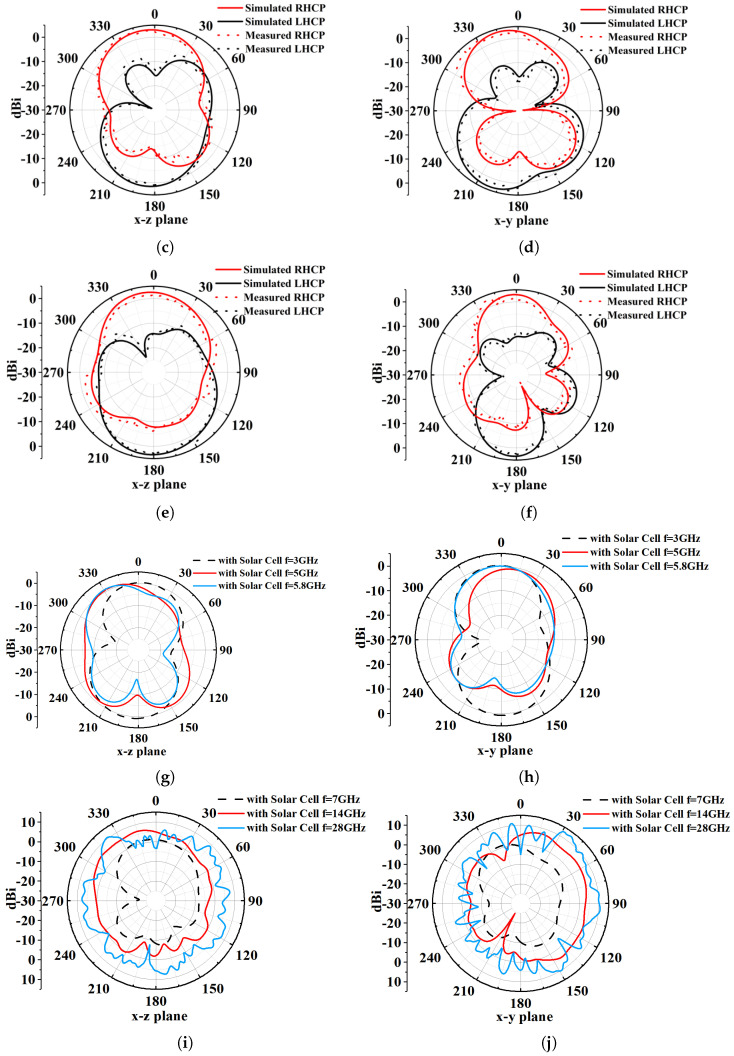
Simulated and measured radiation patterns of antenna (**a**,**b**) at 4.5 GHz, (**c**,**d**) at 5.8 GHz, and (**e**,**f**) at 7.5 GHz; (**g**,**h**) with solar cell at 3 GHz, 5 GHz, and 5.8 GHz; and (**i**,**j**) with solar cell at 7 GHz, 14 GHz, and 28 GHz.

**Figure 14:**
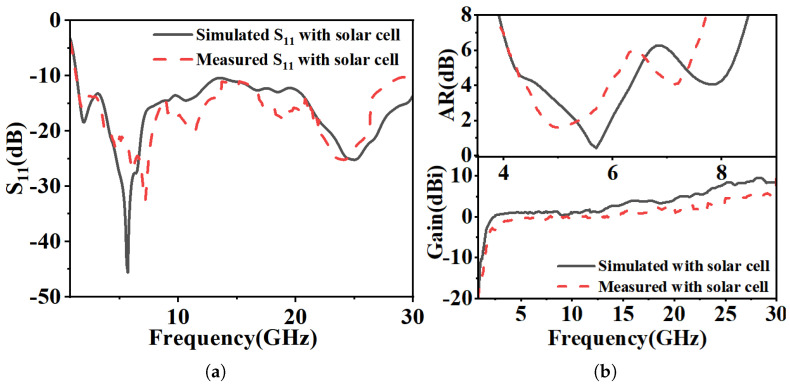
Simulated and measured results with solar cell. (**a**) Return loss. (**b**) Axial ratio and peak gain.

The antenna’s circular polarization (CP) quality was further validated by its radiation characteristics. Figure 12 shows a 3 dB axial ratio beamwidth of approximately 92° at 4.5 GHz for the right-hand circular polarization (RHCP). The radiation patterns in Figure 13 (phi = 0° and phi = 90° cuts) at 4.5, 5.8, and 7.5 GHz confirm stable CP operation within the ARBW, with RHCP dominating in the +z direction and left-hand circular polarization (LHCP) in the −z direction. A slight beam tilt, attributable to the asymmetric slot perturbation, is observed. The impact of solar cell integration (at h_2_ = 2 mm) is shown in Figure 14. While S_11_, axial ratio, and gain are affected, the core performance is maintained. Furthermore, as shown in Figure 13, the antenna’s radiation pattern was characterized across a wider band. At frequencies outside the CP band (e.g., 3, 5, 7, 14, 28 GHz), the antenna exhibits omnidirectional radiation pattern.

### 3.3. Comparison of Antenna Performance

Table 2 represents the comparison between the proposed flexible and transparent wideband CP antenna with its counterparts. The key performance metrics include mechanical adaptability, optical transparency, and polarization characteristics. It is worth mentioning that flexible wideband circular polarized transparent antennas are rarely reported in the existing literature.

## 4. Conclusions

This research involves super-wideband flexible substrate transparent antenna with enhanced axial ratio bandwidth for FG-IoT and hybrid energy harvesting applications. The measurement results verify that it can cover the wide frequency band from 1.33 to 28.58 GHz that makes it SWBA with broad circular polarization. As a result, this antenna exhibits 182% impedance bandwidth and 66% ARBW, with a broad beamwidth of 92° at 4.5 GHz. This antenna features wideband circularly polarized functionality, a characteristic that sets it apart from other documented transparent and flexible antennas. With 92% transparency and a negligible shadow effect, the antenna preserves 98.3% and 96.2% of the photovoltaic cell’s output voltage and current, respectively. This acute performance establishes its viability for future hybrid energy harvesting systems in terrestrial and orbital applications for low-duty-cycle IoT sensors. The antenna features a compact, lightweight design for easy integration onto surfaces like glass windows and windscreens with minor tuning of parameters. Nevertheless, enhancing the efficiency and gain of the SWTA at lower frequencies is a key area for future research.

## Figures and Tables

**Table 1:** Design parameters of the antenna.

Parameters	Value	Parameters	Value
*W*	45 mm	*L*	45 mm
*h*	0.8 mm	$c_{1}$	9 mm
*g*	1 mm	$w_{1}$	15 mm
$l_{1}$	10.85 mm	$w_{2}$	5 mm
$l_{2}$	5.7 mm	$w_{3}$	19.75 mm
$l_{3}$	22.5 mm	$w_{4}$	6.6 mm
$l_{4}$	5 mm		

**Table 2:** Comparison of proposed antenna with antennas in the literature.

Ref.	Size. ($\lambda_{0}^{2}$)	Freq. (GHz)	$|S_{11}|$ BW (%)	Substrate	Conductive Layer	Peak Gain	CP (ARBW)	Efficiency	Transp. (%)
[1] [2021]	0.56 × 0.37	1.13–1.71	40.8	Glass	Mesh	5.3 dBic	Yes (1.06–1.72)	80%	95
[3] [2023]	0.26 × 0.28	2.9–29.92	164	Flexi-glass	Copper	8.1 dBi	No	90%	63.3
[24] [2022]	0.39 × 0.5	4–8	66.7	Soda-lime-glass	FTO	1.2 dBi	No	>46%	65
[25] [2022]	0.23 × 0.4	2.4–11	128	Soda-lime-glass	FTO/ITO	2 dBi	No	60%	72
[26] [2021]	0.16 × 0.14	1.73–20	168	PET-PVC-PET	ITO	4.12 dBi	No	40%	80.2
[27] [2023]	0.47 × 0.58	3.19–9.30	97.83	PET	AgHT-4	2.49 dBi	No	60%	70
This Work	0.2 × 0.2	1.33–28.52	182	PET	ITO	11.5 dBi	Yes (3.88–7.73)	60%	90

All references use $\lambda_{0}$ (free-space wavelength at the lowest frequency).

## Data Availability

The original contributions presented in the study are included in the article. Further inquiries can be directed to the corresponding author.
